# The microbiome-inflammation-immune axis in oral squamous cell carcinoma: from mechanistic insights to therapeutic perspectives

**DOI:** 10.3389/fimmu.2026.1842459

**Published:** 2026-05-26

**Authors:** Zhongjun Wang, QingYuan Bian, Yue Chu, Wanyue Zhu, Ying Qin, Jiwei Zheng

**Affiliations:** 1 Department of Stomatology, The Affiliated Hospital of Xuzhou Medical University, Xuzhou, China; 2 School of Stomatology, Xuzhou Medical University, Xuzhou, China

**Keywords:** biomarkers, immunoregulation, inflammation, microbial dysbiosis, oral microbiota, oral squamous cell carcinoma, tumor microenvironment

## Abstract

Oral squamous cell carcinoma, the most prevalent malignancy of the head and neck, presents ongoing challenges regarding its molecular mechanisms and clinical management. Current research largely focuses on isolated signaling pathways or specific immune responses, often overlooking the potential contributing role of the microbiota in tumorigenesis. This review proposes the “microbiome-inflammation-immune axis” as an interpretive working hypothesis to elucidate how dysbiosis and microbial interactions may activate host inflammatory responses, trigger pattern recognition receptors and signaling pathways, and remodel the immune microenvironment, thereby potentially facilitating oral cancer progression. Concurrently, this paper emphasizes clinical translation by critically compiling and evaluating relevant clinical analytical indicators—such as peripheral blood inflammatory markers and salivary microbial markers—from the fields of inflammation and microbiology. This provides multidimensional reference points for future disease diagnosis and treatment.

## 1 Introduction

According to the latest 2024 statistical report from the International Agency for Research on Cancer (IARC), head and neck malignancies rank as the sixth most common malignant tumors globally, while oral cancer is the 16th most prevalent malignant tumor worldwide (1), Oral squamous cell carcinoma accounts for approximately 90% of oral cancers (2). Despite advances in understanding the etiology and risk factors associated with oral squamous cell carcinoma, survival rates for oral cancers including oral squamous cell carcinoma have not significantly improved, underscoring the urgent need for novel early detection and treatment approaches (3).

A complex bidirectional relationship exists between inflammation and cancer in the development of oral cancers. Periodontitis, as a prototypical chronic inflammatory oral disease, provides a key example of this relationship (4). Research indicates that periodontitis promotes oral squamous cell carcinoma progression through multiple mechanisms, including the maintenance of chronic inflammation and dysregulation of immune responses (4, 5). On one hand, the chronic inflammatory environment leads to sustained release of pro-inflammatory cytokines, accumulation of reactive oxygen species, and degradation of the extracellular matrix. These factors collectively create a microenvironment conducive to tumorigenesis (6). On the other hand, the immunosuppressive state within the tumor microenvironment further exacerbates periodontal inflammation, forming a vicious cycle (7).

In recent years, advances in detection technologies and deepening understanding of the tumor microenvironment (TME) have revealed a strong association between oral squamous cell carcinoma development and dysbiosis of the oral microbiota (8). Analysis of oral microbiota has shown significant enrichment of *Porphyromonas intermedia* in the oral mucosa, dental plaque, and saliva of oral squamous cell carcinoma patients (9). At the mechanistic level, specific oral microorganisms (e.g., *Fusobacterium nucleatum*, *Porphyromonas gingivalis*) are reported to promote carcinogenesis through multiple pathways, including producing carcinogenic metabolites, activating signaling pathways, and inducing inflammatory responses (10). Animal studies further indicate that microbiota transplantation accelerates oral tumorigenesis, while microbiota clearance delays carcinogenesis (11), holding significant implications for therapeutic breakthroughs in oral squamous cell carcinoma.

**Figure 1:**
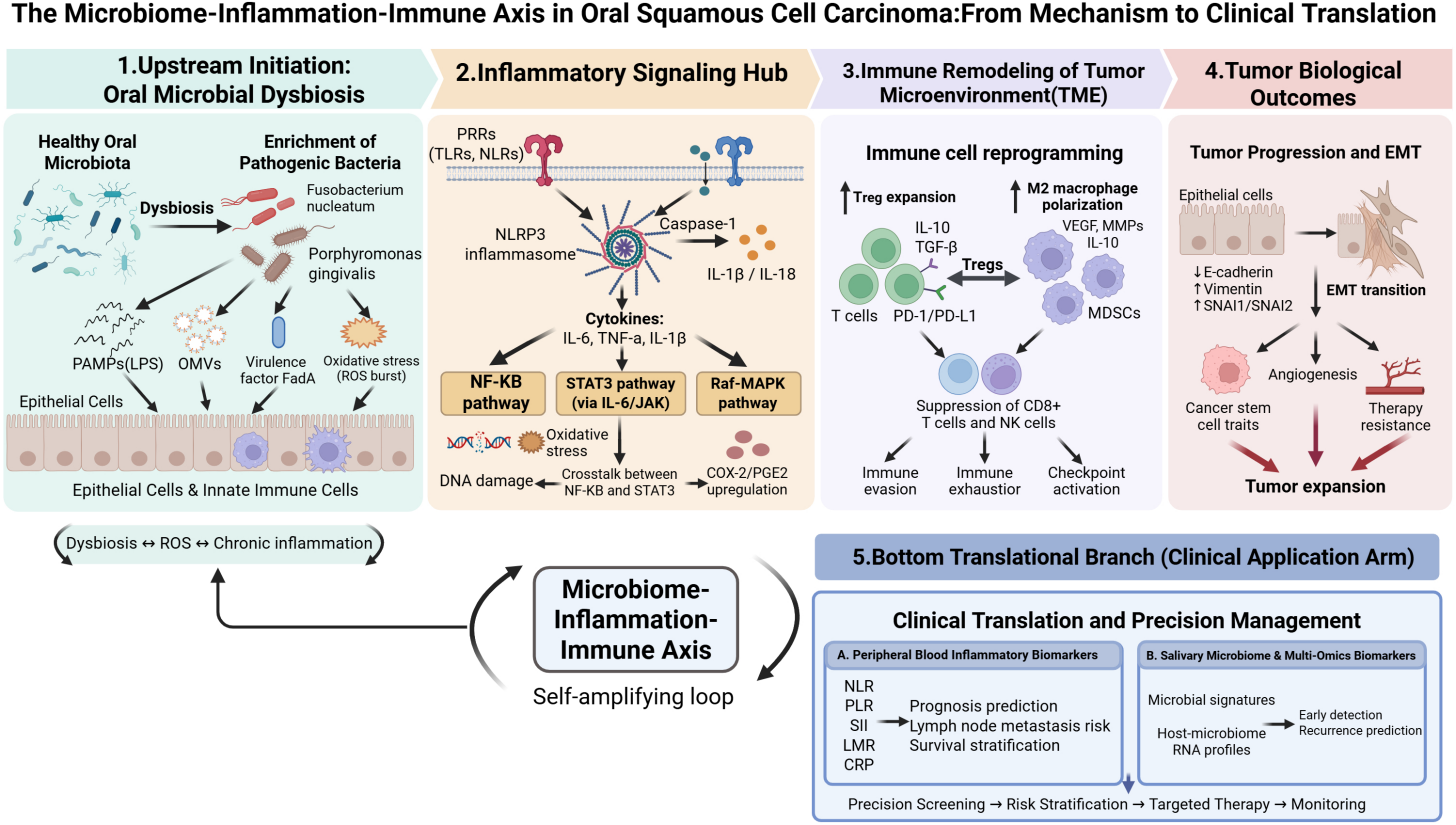
The microbiome-inflammation-immune axis in oral squamous cell carcinoma (OSCC) and its clinical translational implications (1). Upstream Initiation: Oral microbial dysbiosis, characterized by the enrichment of pathogenic bacteria (e.g., *Fusobacterium nucleatum* and *Porphyromonas gingivalis*), triggers local epithelial stress via the release of virulence factors (FadA), pathogen-associated molecular patterns (PAMPs), and outer membrane vesicles (OMVs). (2) Inflammatory Signaling Hub: Pattern recognition receptors (PRRs) activate the NLRP3 inflammasome, leading to the secretion of pro-inflammatory cytokines (IL-1β, IL-6, TNF-α). This triggers downstream cascades, including NF-κB, STAT3, and Raf-MAPK pathways, promoting DNA damage and chronic inflammation. (3) Immune Remodeling of TME: Chronic inflammation drives the expansion of immunosuppressive cells (Tregs, M2 macrophages, and MDSCs) and upregulates inhibitory checkpoints (PD-1/PD-L1), severely suppressing the cytotoxic functions of CD8+ T cells and NK cells. (4) Tumor Biological Outcomes: The inflammatory and immunosuppressive microenvironment promotes epithelial-mesenchymal transition (EMT), enhancing cancer stem cell traits, angiogenesis, and therapy resistance, ultimately facilitating tumor expansion. (5) Clinical Translation: A self-amplifying loop exists among dysbiosis, oxidative stress, and chronic inflammation. Systemic reflections of this axis, notably peripheral blood inflammatory biomarkers (e.g., NLR, PLR, SII, CRP) and salivary multi-omics, serve as robust, non-invasive tools for precision screening, risk stratification, targeted therapy formulation, and prognostic monitoring in OSCC patients. OSCC, oral squamous cell carcinoma; PAMP, pathogen-associated molecular pattern; OMV, outer membrane vesicle; PRR, pattern recognition receptor; TNF, tumor necrosis factor; NF-κB, nuclear factor kappa B; STAT3, signal transducer and activator of transcription 3; MAPK, mitogen-activated protein kinase; TME, tumor microenvironment; Treg, regulatory T cell; MDSC, myeloid-derived suppressor cell; EMT, epithelial-mesenchymal transition.

However, the complex crosstalk among the microbiota, inflammatory responses, and the immune system, particularly their upstream-downstream mechanistic links, remains incompletely understood. This review therefore aims to elucidate how microbiota may act as upstream triggers of the inflammatory microenvironment, how inflammation is hypothesized to shape tumor-immunosuppressive microenvironments through signaling pathways, and how these processes are associated with tumorigenesis. We propose the ‘microbiome-inflammation-immune axis’ as an interpretive working hypothesis to systematically conceptualize these interactions. Unlike previous reviews that treat these domains in isolation, the primary novelty of our model lies in its OSCC-specific, upstream-midstream-downstream hierarchical organization: upstream oral microbiome dysbiosis, midstream activation of inflammatory signaling pathways, and the downstream formation of an immunosuppressive TME. Building upon this defined biological axis, we further review its translational significance by connecting these mechanistic insights to accessible clinical readouts, specifically circulating inflammatory markers and salivary biomarkers. The overall framework, encompassing both the pathogenetic mechanisms and their clinical implications, is illustrated in **Figure 1**.

## 2 Microbiome-inflammation-immunity axis

This section introduces the ‘Microbiome-Inflammation-Immunity Axis’ as an interpretive working hypothesis for oral squamous cell carcinoma pathogenesis. This proposed axis is hypothesized to originate from dysbiosis of the oral microbiome, potentially serving as an upstream trigger. By releasing inflammatory cytokines and pathogen-associated molecular patterns (PAMPs), it is thought to persistently activate the host’s innate immune response, contributing to the establishment and maintenance of a chronic inflammatory state. The inflammatory microenvironment is hypothesized to contribute to the malignant transformation of epithelial cells by modulating diverse intracellular signaling pathways, which may subsequently facilitate the remodeling of the TME toward an immunosuppressive state, potentially contributing to tumor progression and immune evasion.

### 2.1 Upstream trigger: dysbiosis of the oral microbiome

Approximately 30 trillion bacterial cells reside within or on every human body, collectively termed the microbiome. Numerous studies have linked alterations in microbial communities to systemic diseases such as allergies, diabetes, inflammatory bowel disease, and atherosclerosis (3). The oral cavity harbors up to 1,000 distinct microbial species, including bacteria, fungi, and viruses, thriving within a highly dynamic microenvironment (12). This microenvironment is not uniform; rather, it comprises distinct ecological compartments: surfaces (e.g., teeth and dental plaque), within tissues (the oral mucosa), and fluid (saliva).Microbes rarely exist as isolated species but almost always form communities (13). The interdependence among normal oral flora and between these microbes and their host collectively constitutes the oral ecosystem. Many normal flora maintain a dynamic equilibrium with their hosts, which is crucial for preserving host health and serves as one of the body’s non-specific immune factors. However, this balance can be disrupted by risk factors such as smoking, alcohol consumption, and poor oral hygiene, leading to dysbiosis (14, 15). Importantly, a clear distinction must be made between these microbial states. Within the context of this review, “dysbiosis” refers to a compositional and functional imbalance of the resident commensal flora without necessarily invading host tissues, whereas “infection” involves active tissue invasion and pathogenesis driven by specific opportunistic or exogenous pathogens.

Oral dysbiosis manifests primarily as reduced diversity, enrichment of specific pathogens, and a shift in bacterial metabolism from symbiotic to pathogenic pathways. Although studies indicate similar α-diversity in oral microbiota between oral squamous cell carcinoma patients and healthy controls, significant differences exist in β-diversity of microbial composition (16). Specifically, Soyoung Kwak et al. observed a reduction in common symbiotic bacteria in healthy mouths (e.g., *Streptococcus sanguinis*) among oral squamous cell carcinoma patients, alongside increased levels of *Prevotella salivari*, *Streptococcus sanguinis*, *Spirochaeta*, and members of the *β- and γ-Proteobacteria phyla* (17). Furthermore, multiple studies consistently report characteristic enrichment of *Prevotella crispatus*, the Bacteroidetes family, and the Vibrionaceae family (16), along with anaerobic Streptococcus species, *Bacteroides defectivus*, and *Fusobacterium nucleatum* in oral squamous cell carcinoma populations (18).

Dysbiosis of the oral microbiota produces pro-inflammatory cytokines (such as IL-1β, IL-6, and TNF-α), chemokines, and PAMPs, ultimately leading to chronic inflammation, oxidative stress, and the creation of an immunosuppressive microenvironment. Among enriched pathogens, *P. gingivalis* and *F. nucleatum* play particularly prominent roles (19). Studies indicate these bacteria produce bacterial exosomes (bEVs) called outer membrane vesicles (OMVs), releasing virulence factors that interact with the host immune system (20). For example, OMVs from *P. gingivalis* induce macrophages to produce large amounts of inflammatory mediators including TNF-α, IL-12p70, IL-6, IL-10, and IFN-β;*Corynebacterium nucleatum* also upregulates NLRP3 inflammasome and related cytokines (IL-1β, IL-18, IL-6, TNF-α) (21). Furthermore, the *F. nucleatum* adhesin FadA, acting as a pro-inflammatory virulence factor, not only increases the expression of IL-1β, IL-6, and IL-8 but also binds to the host protein PEBP1 to activate the Raf1-MAPK and IKK-NF-κB signaling pathways, thereby further amplifying inflammation (22).

**Figure 2:**
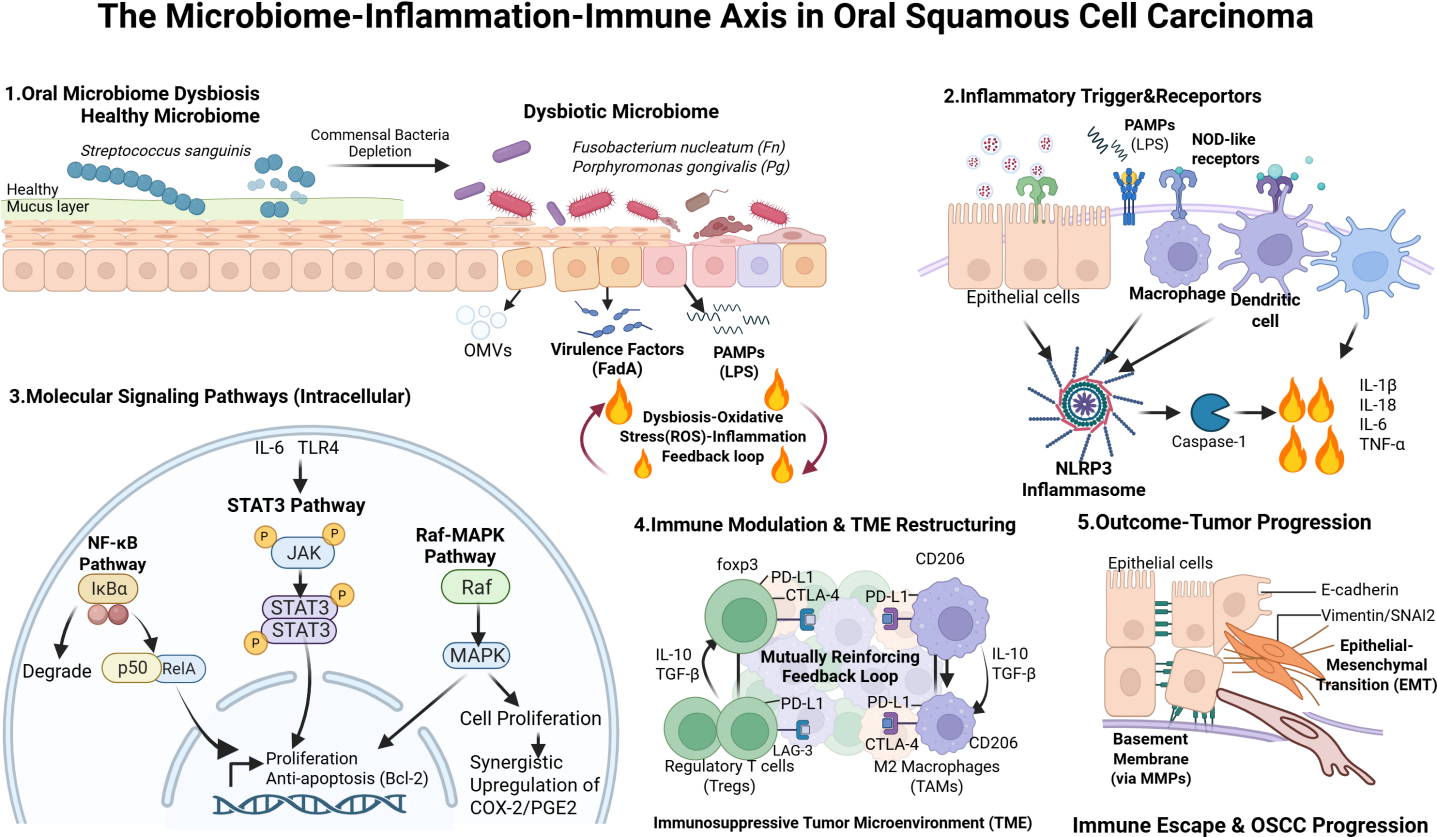
The microbiome-inflammation-immune axis in oral squamous cell carcinoma. (1) Oral Microbiome Dysbiosis: The ecological shift in the oral microbiome is characterized by the depletion of commensal bacteria (e.g., *Streptococcus sanguinis*) and the overgrowth of periodontopathic pathogens, including *Fusobacterium nucleatum* F. nucleatum and Porphyromonas gingivalis (Pg). These dysbiotic microbes release outer membrane vesicles (OMVs), virulence factors (e.g., FadA), and pathogen-associated molecular patterns (PAMPs, such as LPS). (2) Inflammatory Trigger & Receptors: These microbial factors bind to Toll-like receptors (TLRs) and NOD-like receptors (NLRs) on epithelial cells, macrophages, and dendritic cells. This triggers the assembly of the NLRP3 inflammasome and activates Caspase-1, leading to the massive release of pro-inflammatory cytokines (IL-1β, IL-18, IL-6, and TNF-α). Consequently, a vicious feedback loop is established among dysbiosis, oxidative stress (ROS), and chronic inflammation. (3) Intracellular Molecular Signaling: The inflammatory cascade activates multiple intracellular pathways. The NF-κB pathway is activated via IκBα degradation and p50/RelA nuclear translocation; the STAT3 pathway is triggered by membrane receptors (IL-6R, AhR, TLR4) via JAK kinases; and the Raf-MAPK pathway is concurrently engaged. These pathways synergistically drive cellular proliferation, anti-apoptosis (Bcl-2), and the upregulation of COX-2/PGE2. (4) Immune Modulation & TME Restructuring: Persistent inflammation drives the formation of an immunosuppressive tumor microenvironment (TME). A mutually reinforcing feedback loop forms between Foxp3+ Regulatory T cells (Tregs) and CD206+ M2 Macrophages (TAMs) via the secretion of IL-10 and TGF-β, accompanied by the high expression of immune checkpoints (PD-L1, CTLA-4, LAG-3). (5) Outcome - Tumor Progression: This highly inflammatory and immunosuppressive niche ultimately promotes the Epithelial-Mesenchymal Transition (EMT) of OSCC cells. The downregulation of E-cadherin and upregulation of Vimentin/SNAI2 lead to the loss of cellular adhesion, allowing cancer cells to degrade the basement membrane via matrix metalloproteinases (MMPs), facilitating immune escape and tumor progression. OSCC, oral squamous cell carcinoma; Fn, *Fusobacterium nucleatum*; Pg, *Porphyromonas gingivalis*; OMV, outer membrane vesicle; PAMP, pathogen-associated molecular pattern; LPS, lipopolysaccharide; TLR, Toll-like receptor; NLR, NOD-like receptor; TNF, tumor necrosis factor; ROS, reactive oxygen species; NF-κB, nuclear factor kappa B; STAT3, signal transducer and activator of transcription 3; AhR, aryl hydrocarbon receptor; MAPK, mitogen-activated protein kinase; COX-2, cyclooxygenase-2; PGE2, prostaglandin E2; TME, tumor microenvironment; Treg, regulatory T cell; TAM, tumor-associated macrophage; IL, interleukin; TGF-β, transforming growth factor beta; PD-L1, programmed death-ligand 1; CTLA-4, cytotoxic T-lymphocyte-associated protein 4; LAG-3, lymphocyte-activation gene 3; EMT, epithelial-mesenchymal transition; MMP, matrix metalloproteinase.

Dysbiosis and oxidative stress can form a mutually reinforcing vicious cycle, significantly exacerbating local inflammatory responses. Oxidative stress serves as a pivotal link between oral dysbiosis and inflammatory reactions (23). Oxidative stress occurs when the production of ROS exceeds the capacity of antioxidant defense systems. Under oxidative stress conditions, dysregulated immune signaling pathways intensify inflammatory responses (24). Research indicates that ROS possess direct pro-inflammatory effects; excessive ROS induce cellular damage, impair normal physiological functions, trigger the release of inflammatory mediators, and exacerbate inflammatory responses (25). Although the underlying mechanisms remain incompletely understood, existing research suggests that dysbiosis may promote the proliferation of proteolytic Gram-negative bacteria by creating a local anaerobic environment rich in tissue degradation products (26). These bacteria and their metabolites further exacerbate ROS production, forming a positive feedback loop of “dysbiosis-oxidative stress-inflammation amplification” (27). The comprehensive molecular mechanisms detailing how this microbial dysbiosis and oxidative stress trigger downstream intracellular signaling cascades and TME restructuring are illustrated in **Figure 2**.

Oral dysbiosis leads to massive release of PAMPs. These PAMPs are recognized by pattern recognition receptors (PRRs) in the host innate immune system, primarily including Toll-like receptors (TLRs), RIG-I-like receptors, NOD-like receptors, and C-type lectin receptors (28). PAMPs play a crucial role in oral squamous cell carcinoma, particularly through pathways such as NLRP3 inflammasome activation, participating in inflammatory regulation, tumor microenvironment formation, and the balance between cell proliferation and apoptosis. The mechanisms involved are complex and bidirectional (29).

The NLRP3 inflammasome is activated by recognizing PAMPs, thereby promoting the maturation and secretion of IL-1β and IL-18 via caspase-1. These proinflammatory factors enhance chronic inflammatory responses in oral squamous cell carcinoma, induce DNA damage, and may facilitate malignant transformation of epithelial cells. Furthermore, IL-1β released upon NLRP3 activation recruits immunoregulatory T cells and M2 macrophages (30), suppressing antitumor immune responses while promoting angiogenesis and matrix remodeling to support tumor growth (29).

### 2.2 Midstream regulatory hub: activation of inflammation-related signaling pathways

Bacterial secretions may activate inflammatory pathways, leading to a chronic inflammatory state in the tumor microenvironment (19). Key inflammatory pathways include the NF-κB signaling pathway, STAT3 signaling pathway, and Raf1-MAPK pathway.

#### 2.2.1 NF-κB signaling pathway

As a key transcription factor, the NF-κB signaling pathway activates genes involved in immune responses, inflammation, cell growth, and survival. Its abnormal activation is closely linked to the pathogenesis of various inflammatory diseases and cancers (31). Georgios Kamperos et al. observed a progressive increase in the intensity and total score of NF-κB cytoplasmic immunostaining from normal mucosa to oral squamous cell carcinoma, indicating NF-κB activation in the early stages of oral cancer development (32). This activation process is typically initiated by PAMPs (e.g., LPS) and proinflammatory factors (e.g., TNF-α) released from dysbiotic oral microbiota via TLRs or cytokine receptors (19). The classical pathway is typically activated by proinflammatory factors like TNF-α, IL-1β, and LPS, involving IKK complex-mediated degradation of IκBα, thereby releasing the p50/RelA dimer into the nucleus to regulate target gene expression (33).

Persistent activation of the NF-κB signaling pathway leads to massive production of proinflammatory factors, cytokines such as IL-1β, IL-6, and TNF-α, as well as ROS, collectively forming a pro-cancerous inflammatory microenvironment (31, 34). This environment not only serves as a signaling source for cellular proliferation and survival but also directly compromises genomic stability. Jung-Min Oh et al. demonstrated that infections by oral pathogens, notably *F. nucleatum* and *P. gingivalis*, significantly induce elevated expression of the host cell DNA double-strand break marker γH2AX (35). These bacteria or their metabolites (e.g., LPS from *P. gingivalis*) are potent ROS inducers themselves, while inflammatory mediators further amplify oxidative stress, leading to widespread DNA oxidative damage (35).

However, NF-κB signaling regulation is not unidirectional. Notably, IκBα, a key cytoplasmic inhibitor of NF-κB, exhibits an unexpected pro-oncogenic function in oral squamous cell carcinoma. Recent studies demonstrate that IκBα promotes carcinogenic progression by directly interacting with cathepsin B (CTSB) to mediate stemness and epithelial-mesenchymal transition (EMT) characteristics in oral squamous cell carcinoma (36). This suggests that under specific pathological conditions in oral squamous cell carcinoma, IκBα may deviate from its classical inhibitory role and instead participate in maintaining abnormal positive feedback loops of tumor-promoting inflammation and oncogenic signaling, enabling sustained and transformed inflammatory signaling.

Furthermore, NF-κB promotes tumor cell proliferation and anti-apoptosis by upregulating genes such as Cyclin D1 and Bcl-2, while enhancing tumor invasion and metastasis through induction of EMT-associated transcription factors (e.g., Twist, Snail) and matrix metalloproteinases (e.g., MMP-9) (37). NF-κB regulates macrophage polarization, T cell function, and metabolic reprogramming in the tumor microenvironment (38), creating an immunosuppressive environment. Research by Yuhan Wang et al. revealed the role of the NF-κB signaling pathway in the malignant transformation of OSCC. It not only exacerbates inflammatory responses but also enhances cellular survival by upregulating anti-apoptotic proteins such as members of the Bcl-2 family, promoting epithelial cell proliferation and anti-apoptosis (39).

In summary, NF-κB serves as a pivotal molecular bridge linking chronic inflammation to cancer initiation and progression. Activated by stimuli including pathogens, inflammatory cytokines, and PAMPs, it coordinates inflammatory cascades, regulates tumor cell proliferation, survival, and invasion, and facilitates immune-metabolic reprogramming within the tumor microenvironment—collectively contributing to tumor development and progression.

#### 2.2.2 STAT3 signaling pathway

The Signal Transducer and Activator of Transcription (STAT) family comprises cytoplasmic transcription factors including STAT1, STAT2, STAT3, STAT4, STAT5a, STAT5b, and STAT6. These proteins activate upon cytokine and growth factor stimulation, mediating diverse intracellular signaling pathways (40). Signal Transducer and Activator of Transcription 3 (STAT3) is a transcription factor in the Janus kinase (JAK)/STAT signaling pathway (41), It exhibits constitutive activation in human malignancies and is implicated in nearly all hallmark features of cancer, including tumor proliferation, metastasis, angiogenesis, immune suppression, tumor inflammation, metabolic reprogramming, drug resistance, and cancer stemness (42).

During oral squamous cell carcinoma development, STAT3 is activated by upstream cytokines, inflammatory mediators, and growth factor receptors—including IL-6—as well as non-receptor tyrosine kinases (NRTKs) such as JAK and Src family kinases (SFK) (43, 44). Research indicates that in OSCC, cytokines such as IL-6 within the chronic inflammatory microenvironment activate JAK kinases via the JAK-STAT3 pathway. This leads to the phosphorylation and activation of STAT3, promoting transcription of genes associated with proliferation, survival, and angiogenesis while inhibiting apoptosis, thereby contributing to tumorigenesis.STAT3 also participates in regulating tumor stem cell properties, immune evasion, and epithelial-mesenchymal transition, thereby facilitating oral squamous cell carcinoma progression (45). Beyond classical tyrosine and serine phosphorylation activation, STAT3 can also be activated via acetylation, mediating its diverse biological functions (43).

Furthermore, drawing upon findings from various contexts, we propose a hypothetical link wherein metabolites produced by altered microbiota may indirectly activate the STAT3 pathway, thereby potentially contributing to oral squamous cell carcinoma development. Specifically, during oral dysbiosis, certain bacterial metabolites convert tryptophan into indole compounds such as kynurenine precursor (Kyn) (46). These metabolites are hypothesized to serve as ligands for AhR. According to this inferential connection, the aryl hydrocarbon receptor (AhR) activation induces the expression of IDO1 (indoleamine 2,3-dioxygenase 1), which further metabolizes tryptophan into kynurenine, potentially forming a positive feedback loop (47). Moreover, evidence from parallel studies suggests that upon activation, AhR influences MyD88 expression via the TLR4 signaling pathway, thereby indirectly regulating STAT3 activity (48). It should be noted that while this integrated AhR/IDO1/STAT3 axis is biologically plausible, it currently represents an inferred network drawn from different contexts, requiring direct experimental validation specifically within the OSCC microenvironment.

The hyperactivated state of STAT3 regulates the transcription of downstream target genes, playing a key oncogenic role in the initiation and progression of oral tumors. Research by Elina Khatoon et al. indicates that during the multistep progression of oral cancer, the STAT3 pathway plays a crucial role in numerous biological functions, including cancer cell proliferation and survival, immune evasion, angiogenesis, dysbiosis, EMT, invasion, metastasis, autophagy, chemotherapy resistance, and radiation resistance. Specifically, Bickett et al. demonstrated that STAT3 signaling modulates cytokine production within the TME to facilitate the recruitment of immunosuppressive cells, particularly regulatory T cells, thereby driving the formation of an immunosuppressive microenvironment (49).

In summary, STAT3 serves as a pivotal molecular bridge linking dysbiosis to cancer initiation and progression. Activated by stimuli such as cytokines, inflammatory mediators, and dysbiosis, it collectively contributes to tumor development and progression through abnormal cell proliferation, immune evasion, and the formation of an immunosuppressive TME.

#### 2.2.3 Raf-MAPK pathway

The MAPK pathway is a signaling cascade linking extracellular signals to cellular processes such as differentiation, proliferation, and growth. Dysregulation of the MAPK pathway is observed in multiple cancers, particularly with high-frequency activating mutations in RAS and RAF proteins (50). RAF kinase serves as a primary mediator in the MAPK pathway, responsible for the sequential activation of downstream targets that govern diverse cellular and physiological processes. These include organismal development, cell cycle control, proliferation and differentiation, survival, and cell death (51, 52). Defects in this signaling cascade are associated with diseases such as cancer.

No direct experimental evidence currently demonstrates that oral dysbiosis leads to activation of the Raf-MAPK signaling pathway. However, gut dysbiosis causes impaired intestinal barrier function and altered metabolites, which can directly activate oncogenic MAPK-related signaling pathways in colonic epithelial cells (53, 54). This research provides guidance for studying microbial-inflammatory mechanisms in oral squamous cell carcinoma. The activated Raf-MAPK pathway enhances cell proliferation, migration, and angiogenesis (19). Within the tumor microenvironment associated with oral microbiota, these diverse signaling pathways do not operate in isolation. For instance, NF-κB and STAT3 share common upstream drivers and are frequently activated simultaneously, forming a synergistic signaling network (55). Concurrently, these converging pathways (including Raf-MAPK and NF-κB) collaboratively upregulate critical inflammatory mediators, most notably cyclooxygenase-2 (COX-2) and its downstream product PGE2. By acting as central effector molecules, the COX-2/PGE2 axis establishes positive feedback loops that amplify the inflammatory milieu, collectively driving cancer progression (19, 56).

### 2.3 Downstream functional state: formation of an immunosuppressive tumor microenvironment

Oral microbiota, particularly pathogens like *F. nucleatum* and *P. gingivalis*, generate toxic metabolites, secrete cytokines and inflammatory mediators, and activate host signaling pathways through the aforementioned processes. This is thought to contribute to the establishment of an immunosuppressive TME (55), thereby potentially facilitating tumorigenesis. Generally, this immunosuppressive microenvironment comprises cellular and soluble components, and its crucial role in the initiation, development, and progression of OSCC is widely recognized (57, 58). Various cells within the TME, including myeloid-derived suppressor cells (MDSCs), tumor-associated macrophages (TAMs), tumor-associated fibroblasts (CAFs), T lymphocytes, tumor-associated neutrophils (TANs), and dendritic cells (DCs) form a complex and crucial cellular network by secreting cytokines and metabolic byproducts, regulating oral squamous cell carcinoma proliferation, invasion, migration, and angiogenesis (57, 59). The formation of an immunosuppressive tumor microenvironment primarily involves cellular malignant transformation, EMT, and supportive factors associated with tumor progression.

#### 2.3.1 Treg expansion and activation

Regulatory T cells (Tregs) constitute an inhibitory T cell subset that maintains the balance between immune activation and tolerance, playing a pivotal role in immune homeostasis regulation (60). The tumor microenvironment of oral squamous cell carcinoma represents a complex immunological landscape where Tregs serve as central modulators of immunodynamics, playing vital roles in immune suppression and evading immune surveillance (61, 62). Specifically, they exert these suppressive functions by secreting immunosuppressive cytokines such as IL-10,TGF-β, and IL-35, not only impair dendritic cell (DC) maturation and antigen presentation, inducing a tolerogenic DC phenotype, but also suppress the cytotoxic function of CD8^+^ cytotoxic T lymphocytes (CTLs) and natural killer (NK) cells while diminishing the proinflammatory activity of M1 macrophages (62).

Oral dysbiosis and the resulting chronic inflammatory microenvironment significantly promote Treg recruitment, expansion, and functional activation. The accumulation of metabolic byproducts like lactate within the tumor microenvironment further enhances Treg stability and immunosuppressive function, thereby facilitating immune escape and disease progression (60).

Preclinical studies suggest that specific oral pathogens, such as *Candida albicans*, have the potential to contribute to the formation of an immunosuppressive microenvironment. This is driven by mechanisms including systemic infection-induced Treg expansion and the activation of the Dectin-1 signaling pathway—a key innate immune receptor crucial for fungal recognition—which promotes Treg infiltration. However, it is crucial to emphasize that these mechanistic insights are predominantly derived from animal models, including extra-oral contexts such as breast cancer (63). Therefore, extrapolating these generalized findings directly to the human OSCC landscape requires caution, as definitive clinical validation remains limited.

Crucially, the interplay between oral dysbiosis, chronic inflammation, and Tregs is heavily mediated through the coordinated upregulation of immune checkpoint molecules. Relevant experiments indicate that chronic oral inflammation and dysbiosis drive Treg accumulation and significantly enhance PD-1/PD-L1 expression in the tumor microenvironment. Alongside the PD-1/PD-L1 axis, immune checkpoint molecules expressed directly on Treg cell surfaces, such as LAG-3 and CTLA-4, negatively regulate APC costimulatory signaling and overall immune function. By combining these suppressive signals, this integrated checkpoint network further enhances tumor immune escape mechanisms and facilitates tumor growth (7, 64).

In addition to checkpoint-mediated suppression, Treg cells directly induce apoptosis in effector T cells and antigen-presenting cells (APCs) by releasing granzyme. Furthermore, they competitively deplete IL-2 in the microenvironment through high CD25 expression, thereby limiting the survival and proliferation of effector T cells.

#### 2.3.2 M2-type TAM differentiation

TAMs are the most extensively infiltrating immune cells in the TME. TAMs exhibit complex and diverse functions, including mediating angiogenesis, promoting tumor invasion and metastasis, and establishing an immunosuppressive microenvironment (65). TAMs comprise multiple subpopulations, primarily categorized into M1 and M2 subtypes based on their differentiation and activity.M1 macrophages exhibit a pro-inflammatory phenotype and exert anti-tumor effects, whereas M2 macrophages display an anti-inflammatory phenotype and act as pro-tumor modulators (66). While it may seem paradoxical that an anti-inflammatory cell drives tumor progression within an inflammation-driven TME, chronic persistent inflammation fundamentally alters the immune landscape. Rather than sustaining a tumoricidal M1 response, the constant barrage of inflammatory signals in the TME—such as IL-6 and high ROS levels—eventually hijacks macrophage plasticity, forcing undifferentiated M0 macrophages to polarize toward the immunosuppressive M2 phenotype.

Furthermore, specific oral dysbiosis actively drives this M2 polarization. For instance, Jiwei Sun et al. observed that injection of *Corynebacterium nucleatum* significantly increased the number of M2 macrophages within tumors. Further studies revealed that *Corynebacterium nucleatum* induces M2 macrophage polarization by activating the AKT/mTOR signaling pathway, inducing autophagy, and subsequently increasing lactate secretion (67, 68).

By adopting this M2 phenotype, TAMs suppress active anti-tumor immunity and promote tissue remodeling, which explains how chronic inflammation ultimately facilitates tumor progression.M2 macrophages have been shown to express co-inhibitory molecules such as PD-L1 (69), and release anti-inflammatory cytokines like IL-10 and TGF-β to promote tumor immune escape (70). The extracellular matrix provides structural and biochemical support for tumor growth but is also considered a critical barrier to tumor cell dissemination. Thus, basement membrane disruption is a key process for tumor cells to escape the local environment. M2-type TAMs achieve tumor cell dissemination by releasing matrix metalloproteinases (MMPs) to degrade the basement membrane and remodel epithelial cell motility (65). Additionally, M2 macrophages release VEGF to induce angiogenesis, recruit Tregs and MDSCs, thereby promoting tumor progression and coordinating tumor development and metastasis (59).

Simultaneously, M2 macrophages and Tregs exhibit a mutually reinforcing relationship, forming a positive feedback loop that significantly enhances the immunosuppressive state of the tumor microenvironment.M2 macrophages secrete interleukin (IL)-10 and TGF-β to promote Treg differentiation and activation, while recruiting regulatory T cells via C-C chemokine ligand 22 (CCL22), thereby establishing an immunosuppressive mechanism. Tregs can induce macrophage polarization toward the M2 phenotype by secreting Th2 cytokines such as IL-4 and IL-13 (71, 72).

#### 2.3.3 Epithelial-mesenchymal transition

The process of epithelial cells transforming into motile mesenchymal cells, known as epithelial-mesenchymal transition (EMT), pathologically promotes fibrosis and cancer progression (73). In oral squamous cell carcinoma, microorganisms induce inflammation by producing cytokines and chemokines, thereby promoting EMT, immune evasion, and tumor growth (74). Research by Liuyang Cai et al. confirmed that *Corynebacterium nucleatum* activates the TNFα/NF-κB pro-inflammatory pathway, ECM remodeling, and Wnt signaling through its virulence factor FadA, ultimately inducing SNAI2 (SLUG) expression and promoting EMT (75). Furthermore, *Clostridium perfringens* infection can trigger EMT by activating STAT3 to upregulate EMT genes (76) and by modulating the lncRNA MIR4435-2HG/miR-296-5p/Akt2/SNAI1 signaling pathway (77).

EMT represents the initial stage of oral cancer metastasis, causing epithelial cells to lose intercellular junctions, acquire a mesenchymal phenotype, and enhance migration and invasion capabilities. This process is closely associated with local invasion and regional lymph node metastasis (78). EMT endows cancer cells with stem cell-like properties, forming populations of treatment-resistant cancer stem cells (CSCs) that enhance metastatic potential through the EMT program. Furthermore, EMT involves upregulation of mesenchymal markers (e.g., vimentin) and downregulation of epithelial markers (e.g., E-cadherin), leading to loss of epithelial integrity and acquisition of a mesenchymal morphology. This transformation directly correlates with oral cancer aggressiveness and poor prognosis (79, 80).

The above discussion progressively reveals that during the initiation and progression of OSCC, microbiome dysbiosis is not an isolated event. Instead, it reshapes the immune phenotype of the tumor microenvironment by activating chronic inflammation and specific signaling pathways, thereby forming a continuously amplified, mutually reinforcing “microbiome-inflammation-immunity axis”. This in-depth analysis of the axis not only deepens our understanding of oral squamous cell carcinoma’s pathological mechanisms but, more importantly, tightly links microscopic molecular interactions with macroscopic disease progression. It provides critical theoretical foundations and potential targets for translating mechanistic research findings into actionable diagnostic tools and therapeutic strategies in clinical practice.

## 3 Clinical translation applications

Building on the exploration of the “microbiome-inflammation-immunity axis,” clinical translational research follows two parallel and complementary pathways: first, identifying peripheral blood inflammatory biomarkers that systematically reflect axis activation and patient prognosis; second, developing salivary microbiome biomarkers capable of monitoring oral local microbiota. Importantly, while the oral microbiome occupies distinct physical compartments—such as mucosal tissue surfaces, dental plaque, and saliva fluid—saliva acts as an accessible, aggregate biofluid. Biomarkers derived from saliva can therefore effectively reflect both localized tissue dysbiosis and broader microbial shifts within the oral cavity. These dynamic, quantifiable biomarkers hold promise as core tools for achieving precise risk stratification, early diagnosis, treatment efficacy monitoring, and prognosis assessment in oral squamous cell carcinoma. Furthermore, guided by these biomarkers, interventions targeting key nodes of the “microbiome-inflammation-immune axis”—ranging from microbial modulation and anti-inflammatory therapy to immune remodeling—demonstrate therapeutic prospects shifting from broad-spectrum to precision approaches.

### 3.1 Peripheral blood inflammatory markers

Peripheral blood inflammatory markers primarily include complete blood count (CBC) parameters, acute phase reactants (e.g., C-reactive protein CRP, serum amyloid A-SAA), cytokines (e.g., interleukin-6 IL-6), and procalcitonin (PCT).Among various inflammatory mediators, changes in plasma CRP, albumin levels, and platelet-to-lymphocyte ratio (PLR) and/or neutrophil-to-lymphocyte ratio (NLR) have been widely used to measure systemic inflammatory responses in cancer patients (81). Peripheral blood inflammatory biomarkers (PBIBs) for head and neck cancer include neutrophil-lymphocyte ratio (NLR), lymphocyte-monocyte ratio (LMR), platelet-lymphocyte ratio (PLR), and systemic immune inflammation index (SII) (82).

The mechanistic link between the local tumor microenvironment and these systemic markers involves both soluble factors and vesicular transport. Local chronic oral reactions can alter peripheral blood inflammatory markers through immune cell infiltration and the systemic release of local inflammatory cytokines. Peripheral blood inflammatory markers correlate significantly with immune cell infiltration (e.g., macrophages, lymphocytes) in the oral squamous cell carcinoma tumor microenvironment. These peripheral markers reflect systemic inflammatory states, while chronic inflammation within the local TME influences systemic immune responses via cytokine release (82).Concurrently, local inflammatory factors can induce systemic inflammatory responses via the circulatory system. In addition to soluble cytokines, tumor-derived extracellular vesicles (EVs)—particularly exosomes, which are a specific nanoscale subset of EVs—carry specific protein cargo, transmitting pro-inflammatory signals through the bloodstream. These EVs modulate the function of distant immune cells, leading to alterations in peripheral blood inflammatory markers (83).

Currently, peripheral blood inflammatory markers demonstrate increasing value in oral cancer clinical research. Substantial evidence indicates that systemic inflammatory markers, represented by the NLR and PLR, effectively assess patient prognostic risk, including overall survival, lymph node metastasis, and recurrence tendency. For instance, a retrospective analysis by Ningchang Zhu et al. demonstrated that preoperative NLR, PLR, and aspartate aminotransferase-to-alanine aminotransferase ratio (AAR) hold significant clinical value for the initial screening and diagnosis of HNSCC lymph node metastasis, with AAR emerging as a potential blood-based indicator for assessing cervical lymph node metastasis in HNSCC patient (84). Cristina Valero et al. further confirmed that pre-treatment peripheral blood neutrophils, NLR, and Systemic Inflammation Response Index (SIRI) are the most robust independent predictors of overall survival across all peripheral blood leukocytes (PBLs) in oral squamous cell carcinoma (85). Similarly, Marta Ruiz-Ranz et al. found that preoperative NLR, SII, and LMR may be valuable systemic biomarkers for predicting survival in oral squamous cell carcinoma patients, with NLR being an independent predictor of poor prognosis. Furthermore, systemic inflammatory markers show significant correlation with local lymphocyte infiltration in the TME (86). Notably, Davide Mattavelli et al. identified that extremely low preoperative NLR values in oral squamous cell carcinoma carry negative prognostic implications for survival and recurrence (87) Further studies suggest the prognostic value of inflammatory markers may be influenced by HPV infection status. Pardis Noormohammadpour et al. further confirmed that systemic inflammatory biomarkers are associated with survival outcomes in head and neck squamous cell carcinoma patients, with SIR markers potentially exerting a greater impact on survival in HPV-positive HNSCC (88).In lip cancer, Zhilin Li et al. identified NLR as a promising blood biomarker in lip cancer patients, associated with lower survival rates (82).

In summary, systemic inflammatory markers demonstrate significant potential for clinical translation in risk stratification, lymph node metastasis prediction, and prognostic assessment of OSCC. However, several hurdles must be overcome before their routine clinical implementation. First, substantial inter-study variability exists regarding the clinical efficacy of various markers. While NLR is relatively established as a robust indicator of OSCC progression and nodal involvement, consensus remains elusive for other indices. Notably, markers yielding positive results in univariate analyses often lose their independent predictive value in multivariate regression models when adjusted for confounding variables (86). Second, the predictive performance of these markers is heavily modulated by the treatment context. While markers like NLR correlate with therapeutic response in the era of immune checkpoint inhibitors (89), their independent prognostic value in conventional settings—such as surgery or radiotherapy—must be interpreted alongside traditional factors like clinical stage and margin status (90). Furthermore, peripheral blood indicators are susceptible to non-oncological interference, particularly chronic oral inflammations like periodontitis, which can alter the systemic immune milieu and mask the true correlation between inflammation and OSCC progression (91, 92). To address these limitations, future research should prioritize large-scale, multi-center prospective studies to validate these markers across diverse cohorts, particularly focusing on their efficacy in early prediction and distinguishing benign from malignant transformations.

### 3.2 Salivary microbiological markers

Currently, diagnosis of oral squamous cell carcinoma primarily relies on traditional techniques such as biopsy and visual cytology. These methods are invasive, operator-dependent, and subject to diagnostic delays (93). Therefore, there is an urgent need to develop non-invasive, highly sensitive early diagnostic methods. As an easily accessible biological fluid, saliva directly reflects changes in the oral microbiome and the host’s immune status, holding significant importance for early disease diagnosis, improving survival rates, and enhancing prognosis.

In recent years, saliva-based molecular detection technologies have advanced rapidly, demonstrating significant potential particularly in integrating multi-omics information. For example, Banavar et al. recently developed and validated a non-invasive detection method based on salivary macro-transcriptomics—CancerDetect for Oral & Throat cancer™. This study constructed a host-microbiome RNA feature model comprising 270 features (covering 110 microbial species, 72 microbial functional gene clusters, and 88 human genes). In an independent validation cohort, this model achieved 90% sensitivity for OSCC and 84.2% for oropharyngeal squamous cell carcinoma (OPSC), with an overall specificity of 94%. It demonstrated stable discriminatory performance for both early-stage (Stages I/II) and advanced-stage (Stages III/IV) lesions (94). Another study employing non-invasive saliva testing further confirmed the central role of the microbiome in oral squamous cell carcinoma development and progression by analyzing both the microbiome and host gene expression. Hishida et al. found significantly increased abundance of *Fusobacterium* and Bacteroidetes in oral squamous cell carcinoma patients’ saliva, alongside decreased *Streptococcus* abundance; a multi-species diagnostic model demonstrated superior discriminatory power. Crucially, this study revealed a direct link between microbial alterations and clinical prognosis: elevated Bacteroidetes abundance was significantly associated with higher recurrence risk within one year post-surgery (95).

These studies not only validate the clinical feasibility of salivary multi-omics biomarkers for high-precision, non-invasive early diagnosis but also functionally elucidate the synergistic role of microbial activity and host immune responses in oral cancer progression. This provides direct evidence for precision screening and intervention strategies based on the “microbiome-inflammation-immune axis”.

Nevertheless, the dynamic mechanisms governing microbial community changes from precancerous lesions to cancer progression remain incompletely elucidated (96, 97). Despite the immense appeal of salivary biomarkers as non-invasive diagnostic tools for OSCC, translating these findings into routine clinical practice requires overcoming significant methodological and translational hurdles. First, substantial difficulties remain in sample processing and standardization. Salivary composition is highly susceptible to collection time, stimulation methods, storage conditions, and individual physiological states, leading to high sample heterogeneity and a lack of unified standards, which directly compromises the reproducibility and reliability of assay results (98). Second, the current biomarkers often lack sufficient specificity and sensitivity. Many candidate markers can also be elevated in other oral or systemic diseases, thereby increasing the risk of false positives. Furthermore, some studies indicate that their diagnostic sensitivity and specificity remain limited, falling short of the rigorous requirements for early screening (99, 100). Finally, substantial barriers hinder clinical validation and translation. Most studies rely on small sample sizes or single-center data, lacking robust validation from large-scale, multi-center prospective cohorts. Additionally, existing systematic reviews and meta-analyses reveal inconsistent methodological quality across studies, further restricting the translation of these markers into routine clinical care (101). Therefore, while salivary diagnostics hold great promise, they currently represent an area of ongoing research rather than a mature clinical tool.

In summary, non-invasive detection systems represented by peripheral blood inflammatory markers (e.g., NLR, PLR) and salivary multi-omics biomarkers (e.g., microbiota, host RNA) have demonstrated significant potential in early OSCC screening, prognosis prediction, and treatment efficacy monitoring. These biomarkers collectively outline a disease panorama spanning from “local microbe-immune interactions” to “systemic inflammatory responses,” offering new dimensions for precision management of oral squamous cell carcinoma. To address clinical limitations, we must deepen mechanistic research—not only elucidating oral squamous cell carcinoma pathogenesis but also clarifying how key biomarkers regulate the tumor microenvironment. Secondly, we must advance toward integrated and dynamic monitoring. Future studies should focus on developing multimodal diagnostic models that integrate salivary microbiome, peripheral blood inflammatory cells, circulating tumor markers, and radiomics features throughout the entire tumor prevention and treatment process. Finally, we must advance clinical translation and application: design large-scale prospective cohort studies to validate biomarker efficacy in screening and prognosis; develop portable, rapid point-of-care testing technologies; and promote the widespread adoption of salivary microbial and peripheral blood inflammatory biomarkers.

In summary, deepening our understanding of the “microbiome-inflammation-immune axis” is fundamentally reshaping our perception of OSCC pathogenesis and giving rise to a new paradigm of precision diagnosis and treatment centered on non-invasive biomarkers. Despite ongoing challenges, through interdisciplinary collaboration and continuous advancement in integrating fundamental mechanisms with clinical translation, we ultimately aim to achieve early detection, precise intervention, and improved prognosis for OSCC, thereby significantly enhancing patient survival and quality of life.
